# Social Isolation and Loneliness in Alzheimer’s Disease and Related Dementias: Rural and Urban Differences

**DOI:** 10.1002/alz70861_108927

**Published:** 2025-12-23

**Authors:** Ayse Malatyali, Tom Cidav, Rui Xie, Ladda Thiamwong, Lisa Ann Kirk Wiese

**Affiliations:** ^1^ University of Central Florida, 12201 Research Parkway, Suite 300 Orlando, FL USA; ^2^ U.S. Department of Veterans Affairs, Philadelphia, PA USA; ^3^ University of Central Florida, Orlando, FL USA; ^4^ University of Central Florida, Orlando, FL, 32826, FL USA; ^5^ C.E. Lynn College of Nursing, Florida Atlantic University, Boca Raton, FL USA

## Abstract

**Background:**

Approximately one in four older adults in the United States experience social isolation, and many report feeling lonely. Although social isolation and loneliness are associated with an increased risk of cognitive impairment and dementia, it is unclear how this association differs in rural and urban areas. This study examines the differences in the relationship between social isolation and loneliness with cognitive impairment among older adults living in rural and urban areas.

**Method:**

We conducted a cross‐sectional study on Health and Retirement Study participants aged 65 and above (*n* = 2,448) using datasets from the 2020 interviews. Measures were the 27‐item HRS cognition scale, five‐item social isolation scale, UCLA loneliness scale, and Center for Epidemiological Studies Depression scale. Weighted descriptive statistics and logistic regression models were used to assess associations between cognition, loneliness, and social isolation, with stratification by rurality.

**Result:**

Compared to those with normal cognition, older adults with cognitive impairment were significantly lonelier (mean 1.61 vs. 1.49, *p* <0.0001) and more socially isolated (mean 2.83 vs. 2.26, *p* <0.0001). Logistic regression indicated that both loneliness (OR=1.76, 95% CI: 1.38–2.24) and social isolation (OR=1.90, 95% CI: 1.64–2.20) were significantly associated with cognitive impairment, even after adjusting for sociodemographic factors and depression. Rural‐urban stratified analyses revealed that loneliness was significantly associated with cognitive impairment in urban areas (OR=1.72, 95% CI: 1.18–2.51) but not in rural areas. At the same time, social isolation remained significant in both settings, with a stronger effect in rural areas. While men had significantly higher odds of cognitive impairment than women in both rural and urban areas, Black participants in rural areas had notably higher odds of cognitive impairment compared to their White counterparts (OR=5.96, 95% CI: 2.56–13.90).

**Conclusion:**

Loneliness and social isolation are significantly associated with cognitive impairment in older adults, with notable differences by rurality. Tailored interventions addressing psychosocial risks in urban and rural contexts are essential to promote cognitive health in aging populations.

